# Urbanization Level and Woodland Size Are Major Drivers of Woodpecker Species Richness and Abundance

**DOI:** 10.1371/journal.pone.0094218

**Published:** 2014-04-16

**Authors:** Łukasz Myczko, Zuzanna M. Rosin, Piotr Skórka, Piotr Tryjanowski

**Affiliations:** 1 Institute of Zoology, Poznań University of Life Sciences, Poznań, Poland; 2 Department of Cell Biology, Faculty of Biology, Adam Mickiewicz University, Poznań, Poland; Institute of Agronomy, University of Lisbon, Portugal

## Abstract

Urbanization is a process globally responsible for loss of biodiversity and for biological homogenization. Urbanization may have a direct negative impact on species behaviour and indirect effects on species populations through alterations of their habitats, for example patch size and habitat quality. Woodpeckers are species potentially susceptible to urbanization. These birds are mostly forest specialists and the development of urban areas in former forests may be an important factor influencing their richness and abundance, but documented examples are rare. In this study we investigated how woodpeckers responded to changes in forest habitats as a consequence of urbanization, namely size and isolation of habitat patches, and other within-patch characteristics. We selected 42 woodland patches in a gradient from a semi-natural rural landscape to the city centre of Poznań (Western Poland) in spring 2010. Both species richness and abundance of woodpeckers correlated positively to woodland patch area and negatively to increasing urbanization. Abundance of woodpeckers was also positively correlated with shrub cover and percentage of deciduous tree species. Furthermore, species richness and abundance of woodpeckers were highest at moderate values of canopy openness. Ordination analyses confirmed that urbanization level and woodland patch area were variables contributing most to species abundance in the woodpecker community. Similar results were obtained in presence-absence models for particular species. Thus, to sustain woodpecker species within cities it is important to keep woodland patches large, multi-layered and rich in deciduous tree species.

## Introduction

Urbanization is now considered a major driving force of biodiversity loss and biological homogenization, not only in developed countries, but also increasingly in developing ones [1]–[3]. It is a complex process of physical changes that produces a gradient of natural habitat loss that steepens from rural areas toward the urban centre. With urbanization natural vegetation such as forest is left in remnant habitat patches of various sizes and isolation [2] – [5]. These woodland patches are regarded as a key element in maintaining biodiversity in urban areas [6].

Urban-gradient studies have shown that species richness and abundance of many taxa decrease toward centres of urbanization [2], [3]. This decrease is especially apparent for native forest species, while non-native species may benefit from urbanization [3], [7]. A high density of humans in cities is also responsible for a decrease in the abundance of some species that are sensitive to the presence of humans [2]. On the other hand, people living in cities demand contact with natural habitats which provide many services in the urban ecosystem such as human recreation areas, and healthy and aesthetic places [8]. Therefore, finding the balance between two opposing forces: (i) the ever increasing need for more built-up areas in cities and (ii) the need to maintain ecologically diverse systems of woodland patches in cities which provide a number of services for people, is a challenging task [2], [5].

To assess the ecological quality of these woodlands in cities and to successfully protect biodiversity and monitor its changes in urban ecosystems, it is useful to investigate an indicator group of species (i) whose occurrence indicates the presence of a set of other species, (ii) which are keystone species, and (iii) which are sensitive to particular environmental conditions and serve as an early warning of environmental changes. From this perspective, woodpeckers are a valuable group [9]–[11]. Because of their life history they provide cavities for secondary cavity nesters [10] and some woodpeckers may be regarded as keystone species [11]. They are also susceptible to environmental changes caused by different management regimes, thus are regarded as potentially good biotic indicators of forest biodiversity and health [9], [11]–[13]. A few studies have suggested that woodpecker diversity is negatively affected by urbanization, both at the landscape [10], [14] and biogeographical scales [15]. Thus, woodpeckers are possibly good indicators of the urbanization process but this also requires knowledge of how they respond to other environmental factors that change together with urbanization.

The functioning of local populations of woodpeckers in sparsely distributed forest remnants may depend on their sizes and neighbouring habitat patches inhabited by other local populations, as predicted by metapopulation theory [16], [17]. The nature of the matrix surrounding habitat patches may also affect the probability of exchange of individuals between local populations and, thus, occupancy of habitat patches by species [18]–[20]. A matrix of urban land-use may be a greater obstacle for dispersal than a matrix of rural land if animals are less likely to move through an urban matrix than through rural habitats, or if while doing so they experience a higher risk of mortality [21]. Hence, with increasing urbanization one may expect woodland patch area and its composition to have a stronger effect on population occurrence and its size in different species of woodpeckers than the amount and isolation of habitat patches in the surrounding matrix.

In this paper we investigated the effect of urbanization on woodpecker species richness, abundance and community composition in woodland patches within and adjacent to one of the largest city of Poland, Poznan. We hypothesized that (i) species richness and abundance increase with woodland patch area; (ii) the positive effect of woodland patch area on woodpecker populations is stronger in woodland patches surrounded by highly urbanized areas than by more natural landscapes; (iii) variables describing patch suitability for woodpeckers, such as proportion of deciduous tree species or canopy openness are more strongly positively correlated with woodpecker species richness and abundance with increasing urbanization level. In contrast, we also hypothesized (iv) a positive effect of habitat amount in the surrounding of woodland patches on species richness and abundance is stronger in more natural agricultural landscapes than in urban areas. In other words, we expected significant interactions between level of urbanization and a set of variables describing woodland patch composition and its surrounding landscape. We were interested in the effects of the analyzed variables at the level of the entire woodpecker community, as well as the response of individual species.

## Methods

### Ethics Statement

Observation and recording vocal and territorial activity of birds does not require ethics approval or legal permits because it does not involve any important effect on animal welfare. We conducted surveys in woodland patches of forest administrated by The State Forests National Forest Holding which are open to the public, therefore there was no need to ask land managers for approval.

### Study area and plot selection

**Figure 1:**
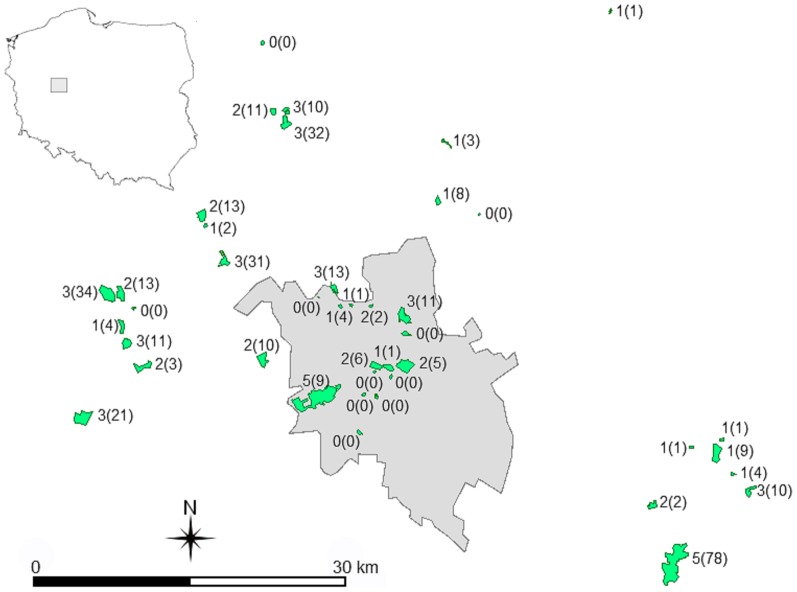
Map of the study area. Green polygons are woodland patches. Shaded area is the city of Poznań. For each woodland patch the number of woodpecker species and their abundance (in brackets) are given.

The study was conducted in Poznań (52°17'–52°30'N, 16°44'–17°04'E) and the surrounding area (up to 32 km from the city centre) in Western Poland in spring 2010 (Fig.1). Poznań is one of the largest Polish cities with 556,000 inhabitants, covering an area of 261 km^2^ (population density 2,123 people per km^2^), and located within Poland's most important agricultural region. Woodlands cover 21% of the area and are mostly patchily distributed within the agricultural matrix and built up areas. Most of woodlands are *Pinus sylvestris* forests, but some, especially along river valleys, are of a semi-natural character, including woodlands with addition of oak (*Quercus* spp.) and ash (*Fraxinus excelsior*). The major land cover types in the surrounding of woodlands are cereal crops and grasslands (44% land cover of the study area) but their cover decreases towards the city centre where human settlements dominates.

Forty two woodland patches were randomly selected (random geographical coordinates in Quantum GIS were selected, then the closest forest patch was taken) from the city and up to 32 km from the city centre (Fig. 1). The selection was stratified with 16 woodland patches selected within administrative boundaries of the city and 26 located outside the city boundary. We ignored patches smaller than 1 hectare or larger than 350 hectares because most of woodlands in the area fall into this range. Very small patches do not have woodpeckers, so inclusion of such small woodland patches would produce meaningless results. Large patches are known as good habitat for woodpeckers and represent the other extreme in terms of size. Therefore, the important issue from a conservation ecology and woodpecker biology perspective is what happens in between these two extreme size categories. The mean distance between woodland patches was 2,388 m (minimum distance between woodland patches was 100 m, the maximum one was 16,300 m).

### Bird surveys

Fieldwork (March–April 2010) covered the peak of vocal and territorial activity of birds associated with establishing and defending territories and mates during the pre-breeding period. Each woodland patch was surveyed twice, with a minimum interval of 30 days between surveys. The survey dates for first surveys varied from 23 March to 31 March and for second surveys ranged from 22 April to 30 April. During each survey the observer moved slowly along transects and recorded the position, behaviour, type of call and number of birds (single, pair or more) and plotted those data onto forest maps according to mapping technique rules [22]. Transects were separated from one another by 100 m and extended to the length of the patch, thus survey time was dependent on patch area but was of not less than 10 minutes duration. In the smallest woodland patches (over 1 ha) a 100 m transect was carried out through the patch from one edge to another. In the middle size woodland patches total transect length was between 5,000 and 6,000 m, and in the largest ones transects were about 26,000 m. The maximum number of persons that conducted surveys where three: ZR, PS and ŁM. Number of persons depended on woodland patch area and the smallest patches where surveyed by one person, the largest ones where divided into three parts each surveyed by one person. Each patch or part of woodland patch was surveyed by different persons during the second survey. We paid special attention to simultaneous records, in order to estimate numbers of unique individuals and hence number of breeding birds. Each suspected case of double counting was treated as just one record in the database.

### Environmental explanatory variables

The following environmental explanatory variables potentially affecting the density of woodpeckers were measured in each woodland:

1. Woodland patch area (ha).
2. Forest cover within 2,000 m from the patch boundary.
3. Canopy openness. Up to 30 photos of the canopy in each woodland patch were made at random points with a digital camera (Nikon Coolpix). All photos were taken in the same manner with the camera aimed vertically at the canopy layer using the default setting of the camera's parameters. Digital photos were then converted into black-white mode in Image J software and the proportion of the white area used as an estimate of canopy openness. The mean from all photos within the patch was used in analyses.
4. Size of trees. The diameters of 10–50 randomly selected trees were measured in each woodland patch at 130 cm above the ground. We only measured trees with a diameter above 10 cm. Trees were measured at randomly chosen sites within a woodland patch. The diameter was calculated from circumference. The number of measured trees was proportional to patch size. The mean diameter per patch was used in analyses.
5. Proportion of deciduous trees. In each patch 1–3 transects 100 m long and 1 m wide were established. All tree species were counted in each transect and the percentage of trees that were deciduous was estimated for each transect and averaged per patch for later analyses.
6. Shrub cover. The percentage cover of the shrub layer in each woodland patch was assessed visually into following categories: 0, 1–5%, 6–10%…95–100%, in 10–50 randomly selected plots (100 m^2^ each) in every woodland patch. Then mean value (calculated from mid-point values of categories) for a patch was used in analyses.
7. Distance of the woodland patch edge to the city centre (taken as the historical central square in the Old City district) was calculated in Quantum GIS.
8. Road density (km per km^2^) within 500 m from the patch boundary was calculated from satellite images using the ImageJ software. Studied woodland patches did not contain roads. We measured surfaced roads. Unpaved roads were omitted because traffic is very low there and it is unlikely to affect woodpecker behaviour.
9. Percentage cover of human settlements within 500 m from the patch boundary was calculated in ImageJ software based on satellite images.

Variables 1–2 describe the fragmentation level of the woodland patches, variables 3–6 describe within-patch conditions and variables 7–9 describe the urbanization gradient. Initially we planned to quantify the amount of dead wood because of their importance for woodpeckers [23]. However, since many forest patches lacked dead wood (the forests being intensively managed), we skipped this variable.

### Data handling and statistical analysis

Analysis was done in two steps: (i) a check for spatial autocorrelation and multicollinearity and (ii) a habitat use analysis with the final/remaining variables. The first analytical goal was to describe the spatial aggregation in species richness and abundance and patch occupancy using Moran's I correlograms [24]. However, since we did not find any significant spatial autocorrelation we used standard statistical tests. We found that three variables (density of roads, cover of human settlements and distance to the city centre) were highly correlated (Table 1). The first principal component from these variables was used as a single measure of urbanization in later analyses. The remaining variables were used unmodified as independent variables because they were only moderately correlated (all r<|0.5|, Table 1) and it is often assumed that regression models are robust to multicollinearity if the correlation between variables is lower than r = |0.6| [25]. All these analyses were run in the SAM 4.0 statistical software [26].

**Table 1:** Pearson correlation coefficients between variables potentially influencing woodpecker species richness, abundance and patch occupancy.

	(1)	(2)	(3)	(4)	(5)	(6)	(7)	(8)	(9)	(10)
(1) Area	-	**−0.371 (0.014)**	−0.220 (0.162)	0.089 (0.571)	−0.038 (0.810)	0.068 (0.665)	**0.320 (0.039)**	**−0.393 (0.010)**	−0.224 (0.153)	**−0.321 (0.036)**
(2) ForCov		-	**0.499 (<0.001)**	**−0.476 (0.001)**	**−0.473 (0.001)**	−0.112 (0.476)	0.134 (0.360)	0.031 (0.845)	−0.124 (0.430)	−0.075 (0.635)
(3) CanOpen			-	**−0.430 (0.009)**	−0.289 (0.063)	−0.073 (0.646)	0.177 (0.260)	0.19 (0.219)	0.149 (0.346)	0.178 (0.254)
(4) Diagonal				-	**0.446 (0.003)**	−0.182 (0.247)	0.216 (0.169)	0.193 (0.219)	0.223 (0.155)	0.216 (0.164)
(5) Deciduous					**-**	−0.025 (0.874)	**−0.474 (0.001)**	**0.422 (0.005)**	**0.493 (0.001)**	**0.475 (0.001)**
(6) Undergrowth						**-**	0.026 (0.868)	0.039 (0.803)	0.011 (0.944)	0.026 (0.866)
(7) DisCentr							**-**	**−0.964 (<0.001)**	**−0.964 (<0.001)**	**−0.878 (<0.001)**
(8) Roads								-	**0.859 (<0.001)**	**0.964 (<0.001)**
(9) Settlement									**-**	**0.934 (<0.001)**
(10) Urban										

Significance values are given in parentheses. Statistically significant correlations are emboldened. Variable codes: Area – woodland patch area, ForCov – cover of forests within 2000 m from the patch boundary, CanOpen – tree canopy openness, Diagonal – mean diameter of trees, Deciduous – percentage of deciduous trees, Undergrowth – mean percentage shrub cover, DisCentr – distance to the city centre, Roads – density of roads within 500 m from the patch boundary, Settlement – percentage cover of human settlements within 500 m from the patch boundary, Urban – urbanization index.

The relationship between environmental variables and woodpecker species richness and abundance (number of individuals) was analyzed using generalized linear models with Gaussian error and identity link-function. We introduced a quadratic term of the variable canopy openness as this explained more variation in woodpecker data than the linear relationship. We used the maximum number of species and individuals recorded in the two counts as estimates of woodpecker species richness and abundance (maximum values were strongly correlated with average values (r = 0.930, P<0.001). In all generalized linear models we built all possible model combinations including a null model with intercept only and models with interactions between urbanization index and other environmental variables. We ranked models according to their ΔAICc values and used the model with the lowest AICc together with associated weight value (the probability that a given model is the best) as that best describing the data. We considered models with ΔAICc lower than 2 as equally good [27]. We used model averaging for estimates of function slopes of parameters of interest [27]. For model averaging we used 99% confidence set (we used all models with a sum of weights equalling 0.99). Thus models with ΔAICc higher than 2 were also included in model averaging (but their ΔAICc were usually lower than 7 thus still had support). Burnham and Anderson [27] have stressed that model averaging should be done with all hypothesized reasonable models instead of using a cutoff (delta AICc<2) in the score differences. Finally, model weights were used to define the relative importance of each explanatory variable across the full set of models evaluated by summing weight values of all models that included the explanatory variable of interest [27].

A canonical ordination was used to relate the abundance of the individual species to eight environmental variables using the CANOCO 4.0 package [28]. Since the length of the longest gradient in detrended canonical correspondence analysis (DCCA) was so short (0.9) we opted to use redundancy analysis (RDA) for this ordination. Species data were *log_e_*(x+1) transformed before analysis. Variables in RDA were introduced by the forward stepwise procedure and their statistical significance (based on P – values) was tested from 499 permutations.

In addition to RDA, we built presence-absence models (i.e. patch occupancy) to calculate the effect of each environmental variable for each species by means of model selection based on ΔAICc values [27]. Presence-absence data were analyzed by generalized linear models with a logit-link function and binomial error variance. We used the approach introduced by MacKenzie et al. [29], [30] that estimates patch occupancy with imperfect detection probability. Presence–absence data and the resulting estimates can be confounded by detection error, namely that a recorded ‘absence’ may in fact be a non-detection of a species rather than a true absence. Using such data with naive estimates will most likely result in underestimates of occupancy [29], [30]. Calculations were performed with the program Presence 3.0 [31].

When necessary, we used log_e_ transformation to reduce the effects of outliers [32]. The decision to transform data was based on Kolmogorov-Smirnov tests. Moreover, in all regression models, variables were standardized to allow a direct comparison of slope (beta) estimates (larger values of betas indicate stronger relationships between explanatory and dependent variables). Model selection for species richness and abundance was performed in the SAM 4.0 statistical software [26] and model selection for presence-absence data was performed with the program Presence 3.0 [31]. All estimates of statistical parameters (means, betas) are quoted with standard errors (SE) and 95% confidence intervals (CI).

## Results

### Species richness and abundance

**Figure 2:**
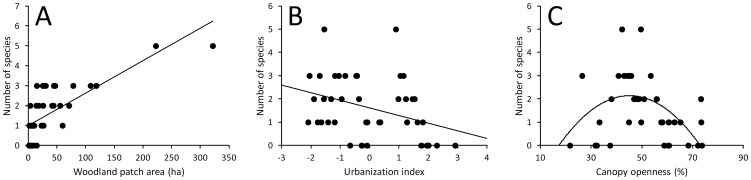
Relationship between woodpecker species richness and (a) woodland patch area, (b) urbanization index and (c) canopy openness (percentage of white area on the canopy pictures).

A total of 6 species and 582 individuals were observed during fieldwork. These were *Dendrocopos major* (87.3% of all individuals), *Dryocopus martius* (6.4%), *Picus viridis* (3.0%), *Dendrocopos minor* (2.3%), *Dendrocopos medius* (0.6%) and *Picus canus* (0.4%). Mean number of species was 1.6±0.2 (range: 0–5) and mean number of individuals was 8.7±2.2 (range: 0–78) per woodland patch. The model selection based on ΔAICc showed that seven models explaining woodpecker species richness were equally good (Table 2). The best models explained 67–70% of variation in woodpecker species richness (Table 2). Explanatory variables that were present in the best models (betas, SE and 95% CI given in brackets are model-averaged estimates across all possible models) were woodland patch area (beta = 1.028±0.139, 95% CI: 0.757–1.300, importance = 1.000, Fig. 2), urbanization index (beta = −0.090±0.031, 95% CI: −0.156–−0.025, importance = 0.355, Fig. 2), percentage of deciduous species (beta = 0.003±0.001, 95% CI: 0.001–0.005, importance: 0.310), shrub cover (beta = 0.071±0.032, 95% CI: 0.008–0.134, importance: 0.242), and quadratic term of canopy openness (beta = −0.256±0.087, 95% CI: −0.427–−0.085, importance = 0.534) with the latter indicating that species richness is the highest at moderate values of canopy openness (Fig. 2).

**Table 2:** Best models describing species richness and abundance of woodpeckers in woodland patches.

No.	Model	k	r^2^	AICc	Δ AICc	*w*
	**Species richness**					
1	Area+CanOpenQ	3	0.67	101.806	0	0.054
2	Area	2	0.68	101.865	0.059	0.052
3	Area+ Deciduous+ CanOpenQ	4	0.70	102.326	0.520	0.041
4	Area+Urban+ CanOpenQ	4	0.70	102.393	0.587	0.040
5	Area+ CanOpen	3	0.68	103.110	1.305	0.028
6	Area+Urban	3	0.68	103.558	1.753	0.022
7	Area+ Undergrowth	3	0.68	103.575	1.769	0.022
	**Abundance**					
1	Area+ Urban+Deciduous+CanOpenQ	5	0.77	128.338	0	0.185

For each model the number of parameters (k), variance explained by the model (r^2^), the Akaike information criterion score (AICc), the difference between the given model and the most parsimonious model (Δ AICc) and Akaike weight (*w*) are listed. CanOpenQ – quadratic term of canopy openness. For explanations of other variable codes: see Table 1.

**Figure 3:**
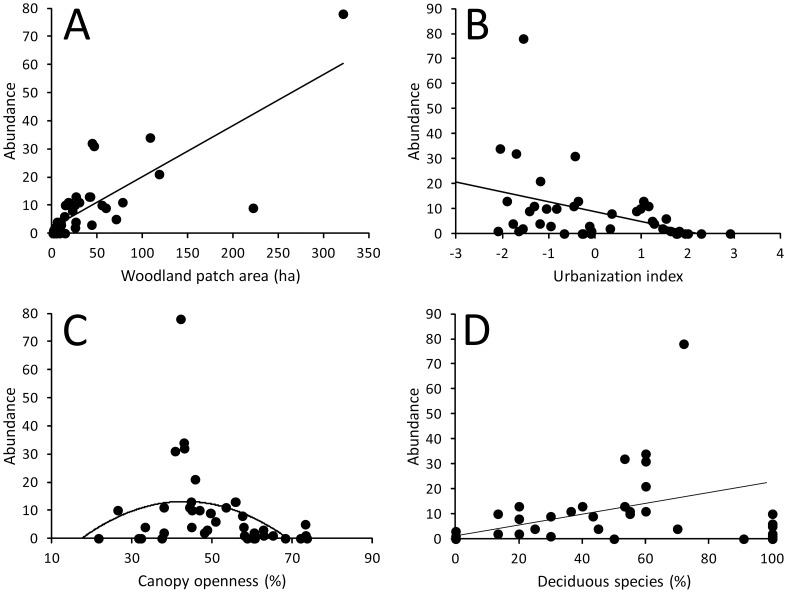
Relationship between woodpecker abundance and (a) woodland patch area, (b) urbanization index, (c) canopy openness (percentage of white area on the canopy pictures) and (d) percentage share of deciduous trees.

One model explaining 77% of variation in woodpecker abundance was the best based on ΔAICc (Table 2). Explanatory variables present in the best model were woodland patch area (beta = 7.692±1.578, 95% CI: 5.992–11.342, importance = 1.000, Fig. 3), urbanization index (beta = −0.266±0.092, 95% CI: −0.446–−0.086, importance = 0.792, Fig. 3), percentage of deciduous species (beta = 0.243±0.082, 95% CI: 0.082–0.404, importance: 0.530, Fig. 3) and quadratic term of canopy openness (beta = −0.292±0.106, 95% CI: −0.500–−0.084, importance: 0.749). This last effect indicated that abundance of woodpeckers was highest at moderate values of canopy openness (Fig. 3).

Given that *Dendrocopos major* was by far the most numerous and widespread species in this study, we also analysed how strongly the distribution patterns of this species in the study area drive the obtained results. When data were analyzed without this dominant species results were very similar. The model selection based on ΔAICc showed that seven models explaining woodpecker species richness were equally good (Table 3). The best models explained 61–66% of variation in woodpecker species richness when *Dendrocopos major* was excluded (Table 3). Explanatory variables that were present in the best models were woodland patch area (beta = 0.801±0.120, 95% CI: 0.628–1.038, importance = 1.000), percentage of deciduous species (beta = 0.229±0.078, 95% CI: 0.077–0.381, importance: 0.495), quadratic term of canopy openness (beta = −0.204±0.068, 95% CI: −0.337–−0.077, importance = 0.405), and shrub cover (beta = 0.116±0.035, 95% CI: 0.047–0.184, importance: 0.318).

**Table 3:** Best models describing species richness and abundance of woodpeckers in woodland patches when the dominant species, *Dendrocopos major*, was excluded from analyses.

No.	Model	k	r^2^	AICc	Δ AICc	*w*
	**Species richness**					
1	Area+Deciduous+CanOpenQ	4	0.66	91.498	0	0.069
2	Area+CanOpen	3	0.63	91.897	0.400	0.056
3	Area	2	0.61	92.104	0.606	0.051
4	Area+Canopy+Deciduous+ CanOpenQ	5	0.67	92.692	1.194	0.038
5	Area+ Deciduous	3	0.62	92.742	1.244	0.037
6	Area+Undergrowth	3	0.62	93.060	1.562	0.032
7	Area+Canopy+ Undergrowth	4	0.64	93.130	1.632	0.030
	**Abundance**					
1	Area+Deciduous+Urban	4	0.66	47.440	0	0.119
	Area+Deciduous+Urban+CanOpenQ	5	0.67	48.666	1.226	0.065
	Area+Deciduous	3	0.62	48.902	1.462	0.057

For each model the number of parameters (k), variance explained by the model (r^2^), the Akaike information criterion score (AICc), the difference between the given model and the most parsimonious model (Δ AICc) and Akaike weight (*w*) are listed. CanOpenQ – quadratic term of canopy openness. For explanations of other variable codes: see Table 1.

Three models explaining woodpecker abundance (without *Dendrocopos major*) were equally good basing on ΔAICc (Table 3). The best models explained 62–67% of variation in the woodpecker abundance (Table 3). Explanatory variables present in the best models were woodland patch area (beta = 0.458±0.073, 95% CI: 0.315–0.602, importance = 1.000), percentage of deciduous species (beta = 0.188±0.070, 95% CI: 0.051–0.325, importance: 0.781), urbanization index (beta = −0.132±0.042, 95% CI: −0.214–−0.050, importance = 0.547), and the quadratic term of canopy openness (beta = −0.090±0.033, 95% CI: −0.155–−0.025; importance: 0.329).

### Factors shaping species composition of woodpecker community

**Figure 4:**
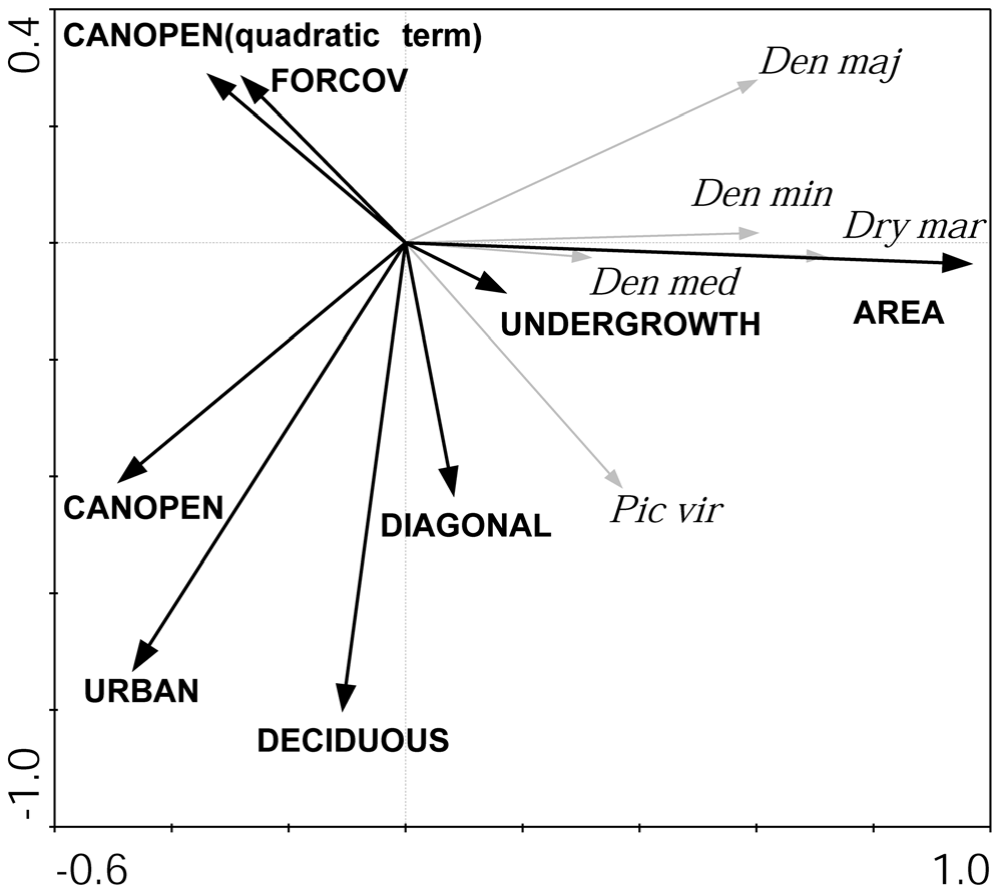
RDA ordination of six environmental variables in relation to woodpecker species in 42 habitat patches. Species are identified by abbreviated scientific names. Labels for species occurring in less than five patches have been omitted. Explanation of variable codes see Tables 1 and 2.

The first two axes of the RDA ordination explained 56.2% of the variation in woodpecker species abundance, of which the environmental variables explained 90.3%. The ordination of the species is shown in Fig. 4 and forward selection of explanatory variables indicated that only woodland area and urbanization index had a statistically significant effect on the woodpecker community (Table 4). All species were positively associated with axis 1. The ordination of the environmental variables suggests that positive values on axis 1 were associated with larger woodland patches and less urbanized landscapes (Fig. 4).

**Table 4:** Results of forward selection of environmental variables explaining patterns in woodpecker community structure in forest patches.

Variable code	F	P
Urban	3.79	**0.010**
Area	2.57	**0.046**
Deciduous	2.11	0.076
ForCov	0.49	0.748
Undergrowth	0.43	0.782
Diagonal	0.34	0.850
CanOpen*	-	-
CanOpenQ*	-	-

The analysis was performed using Monte Carlo tests with 499 permutations. Variables are ordered according to their stepwise inclusion into the model. Significant effects are emboldened. For explanations of variable codes: see Tables 1 and 2.

* - Variables were not included in the stepwise procedure since they did not improve the fit of the model.

### Presence-absence models for individual species

Presence-absence models explained between 21% (*Picus viridis*) and 53% (*Dryocopus martius*) of the variation in the data (Table 5). Detection probability in a patch was high in *D. major* and *Dryocopus martius* but in *Dendrocopos minor* and *Picus viridis* was much lower (Table 6).

**Table 5:** Best models describing patch occupancy of the woodpecker species in woodland patches.

No.	Model	k	r^2^	AICc	Δ AICc	*w*
	***Dendrocopos major***					
1	Area+ Deciduous	2	0.43	29.299	0	0.175
2	Area+ Deciduous+ Urban	4	0.44	31.105	1.806	0.075
	***Dendrocopos minor***					
1	Area+ Urban	3	0.36	34.736	0	0.119
2	Area	2	0.32	35.053	0.316	0.101
3	Area+ Deciduous+ Urban	4	0.38	35.840	1.104	0.068
4	Area+ Undergrowth+ Urban	4	0.38	36.23	1.494	0.056
5	Area+ Undergrowth	3	0.33	36.649	1.912	0.046
	***Picus viridis***					
1	Area+ Deciduous	3	0.24	45.141	0	0.131
2	Area+ ForCov+ Deciduous	4	0.27	46.027	0.886	0.084
3	Area+ Urban	3	0.21	46.593	1.452	0.063
4	Area+ Deciduous+ Urban	4	0.26	46.714	1.573	0.060
	***Dryocopus martius***					
1	Area+ CanOpen	3	0.52	32.879	0	0.120
2	Area	2	0.48	33.504	0.625	0.088
3	Area+ CanOpen+ Deciduous	4	0.53	34.329	1.450	0.058
4	Area+ CanOpen+ Undergrowth	4	0.53	34.377	1.498	0.057
5	Area+ Undergrowth	3	0.49	34.727	1.848	0.048
6	Area+ ForCov+ CanOpen	4	0.52	34.738	1.858	0.047

For each model the number of parameters (k), variance explained by the model (r^2^), the Akaike information criterion score (AICc), the difference between the given model and the most parsimonious model (Δ AICc) and Akaike weight (*w*) are listed. In each model the species detection probability was estimated. No models were built for *Picus canus* and *Dendrocopos medius* because they were present in only one and two forest patches, respectively. For explanations of variable codes: see Table 1.

**Table 6:** Estimation of each model detection probability (*p*), naive estimation of patch occupancy (ψ_r_) and patch occupancy estimated after taking the imperfect detection into account (ψ_d_).

Species	Number of occupied patches	*p*±*SE*	ψ_r_	ψ_d_ ± *SE*
*Dendrocopos major*	32	0.84±0.05	0.76	0.78±0.07
*Dryocopus martius*	13	0.63±0.12	0.31	0.36±0.08
*Dendrocopos minor*	10	0.35±0.16	0.24	0.43±0.21
*Picus viridis*	10	0.33±0.15	0.24	0.42±0.19
*Dendrocopos medius*	2	-	0.05	-
*Picus canus*	1	-	0.02	-

Models with constant detection probability fitted better than survey specific models in all species.

Patch occupancy in *Dendrocopos major* was positively affected by woodland patch area (beta = 2.780±1.005, 95% CI: 0.810–4.750, importance = 0.998) but negatively by percentage of deciduous trees (beta = −0.910±0.432, 95% CI: −1.757–−0.063, importance = 0.695) and urbanization index (beta = −0.803±0.400, 95% CI: −1.587–−0.018, importance = 0.379; Table 5).

Patch occupancy in *Dendrocopos minor* was positively affected by woodland patch area (beta = 2.006±0.715, 95% CI: 0.854–3.776, importance = 0.991), shrub cover (beta = 0.678±0.342, 95% CI: 0.008–1.348, importance = 0.307) and negatively by the urbanization index (beta = −1.145±0.516, 95% CI: −2.343–−0.250, importance = 0.572; Table 5). The percentage of deciduous trees was included in one of the best models, but was statistically non-significant because the 95% CI of the estimate overlapped with zero (beta = 0.374±0.251, 95% CI: −0.118–0.866, importance = 0.300).

Patch occupancy in *Picus viridis* was positively affected by woodland patch area (beta = 1.684±0.666, 95% CI: 0.573–3.283, importance = 0.933) and percentage of deciduous trees (beta = 1.227±0.615, 95% CI: 0.022–2.432, importance = 0.675; Table 5). The urbanization index and forest cover in the landscape, although both included in some of the best models (Table 5), were statistically non-significant (urbanization: beta = 0.913±0.500, 95% CI: −0.010–2.024, importance = 0.406; forest cover: beta = −0.393±0.396, 95% CI: −1.232–0.351, importance = 0.333).

Patch occupancy in *Dryocopus martius* was positively affected by woodland patch area (beta = 4.577±1.757, 95% CI: 2.027–9.216, importance = 1.000) and negatively by canopy openness (beta = −2.055±0.990, 95% CI: −4.611–−0.483, importance = 0.569, Table 5). Shrub cover, cover of forests in the landscape and percentage of deciduous trees were present in some of the best models (Table 5), but were statistically non-significant (shrub cover: beta = −0.830±0.566, 95% CI: −2.099–0.210, importance = 0.320; forest cover: beta = 0.667±0.401, 95% CI: −0.069–1.552, importance = 0.274; percentage of deciduous trees: beta = 0.207±0.605, 95% CI: −1.010–1.471, importance = 0.241).

No model was built for *Picus canus* and *Dendrocopos medius* because they occurred only in one and two woodland patches, respectively.

## Discussion

To the best of our knowledge this study is the first to investigate responses of the woodpecker community to a complex group of anthropogenic and environmental factors. Our study reveals that increasing urbanization and decreasing woodland patch area had a profound negative effect on species richness and abundance of woodpeckers. Moreover, other environmental variables describing within-patch characteristics, such as the percentage of deciduous species in woodland patches, openness of canopy and shrub cover in a woodland patch also affected the woodpecker community. However, environmental variables probably influenced woodpecker species richness and abundance independently of urbanization level because we did not find any effect of interactions between urbanization index and these variables.

Our results are in accordance with general findings that habitat fragmentation (leading to a decrease in the size of habitat patches) and urbanization are among the most important factors influencing wild species communities. Sandström et al. [14] also found negative effects of urbanization on woodpecker species richness. This indicates that urban areas may be an environmental filter [33] that leads to lowered species richness and population sizes of woodpeckers. This may also suggest that woodland patches within highly urbanized areas may be of lower quality than in rural areas and this, eventually, should result in low reproductive success and/or survival as was found in some passerine species inhabiting cities [34]. Relevant studies on woodpeckers are lacking, though. In our study, the urbanization index was derived from three highly correlated variables: distances from the city centre, density of roads and cover of human settlements. Thus, the effect of urbanization on woodpeckers may be also multi-dimensional. First, woodpeckers may avoid areas with high density of human population which may directly disturb the behaviour of birds. The negative effect of urbanization on the woodpecker community may also result from dense road networks. Collisions with cars may be an important factor contributing to mortality [35], [36]. Second, dense road traffic may affect the behaviour of birds and roads may become a behavioural barrier for individuals wanting to cross them [36], [37]. The structure of the landscape around woodland patches in urbanized areas may also be a factor limiting the occurrence of woodpeckers. Many bird species depend on the structure and composition of the matrix in the surrounding landscape [38]. Dense human settlements and blocks of flats may be an inhospitable environment where movements may be impaired. However, there is also the possibility that in these areas woodpeckers can find additional resources provided, for example, in bird feeders, but this applies especially to winter time [7]. In spite of the fact that our research was carried out in a landscape strongly transformed by human activity, six of the seven woodpecker species that potentially could be recorded were found [39]–[41]. This may be because woodland patches within study area are rather well connected with woodlands occurring in the rural area surrounding the city of Poznań and through green zones reaching the city centre, which may function as dispersal corridors for birds and other taxa [14], [42].

The size of habitat patches has usually been regarded as a critical variable in determining their suitability for a given species or entire communities and was also confirmed in our study [43]–[45]. Thus, it has been suggested that conservation of the so called ‘area-sensitive’ species requires protecting large areas of continuous habitat [43]. However, many other bird species do not show that response [44], [46]. Woodpeckers are territorial species thus they require a minimum area to establish territories, and since woodpeckers occupy home-ranges almost year-round it is possible that the species composition is affected by metapopulation dynamics, with smaller woodland patches more often having local extinctions/emigrations resulting in a lower number of individuals and species than in larger woodland patches [6], [47], [48]. Moreover, woodland area may affect breeding biology of species. Mazgajski and Rejt [45] showed that *Dendrocopos major* had lower reproductive parameters in small woodland patches than in larger ones. Thus, further demographic studies on woodpeckers are required to understand how urban ecosystem and habitat patch area affect their biology and lead to a decreasing number of species and abundance. Factors other than urbanization and woodland patch area were also found to be important in explaining woodpecker abundance patterns in our study. They all were related to within-patch characteristics. The percentage of deciduous species in woodland patches, openness of canopy layer and shrub cover affected species richness and abundance of woodpeckers.

Forest with stands of deciduous species support a larger number of woodpecker species than those only of conifers [15], [39]. Moreover, different woodpeckers prefer different tree species and/or of different ages [10], [41], [47], [49]. For example, coniferous stands are favoured by *Dendrocopos major*. Although this species has a wide foraging niche [50], it often specializes in utilization of pinecones, especially in winter [50], [51]. In our study area this species foraged almost exclusively on *Pinus sylvestris* cones, because of extremely low representation of other coniferous species in woodland patches that we studied. We observed only one case of predation on a cone of *Picea abies* and two cases on cones of *Larix decidua*. In contrast, *Picus viridis* occurs mostly in deciduous woodland patches and in cities it is linked with large parks, with open grass as foraging places [10], [11], [40].

We found that species richness and abundance were the highest at moderate values of canopy openness. This result indicates that woodpeckers avoid open spaces in studied woodland patches, contrary to results from Romania [11]. The open spaces within forests may be positively correlated with increased predation risk [52], by such species as *Accipiter gentilis*, the major predator of woodpeckers in Europe [53].On the other hand, very dense forest may possibly hamper movements and foraging, especially in larger woodpecker species.

The positive effect of the shrub layer on woodpecker species richness in our study may be explained by the fact that a multi-layered vegetation structure of woodlands leads to niche partitioning in woodpeckers which is beneficial for species diversity [4], [47], [49] [54]. Some species, such as *Dendrocopos minor* use small branches or forage on young trees while larger species forage on barks of mature trees [54].

### Conclusions and recommendations

Our study suggests that the woodpecker community is an efficient indicator of the negative impact of urbanization on woodland areas. However, we believe from the practical perspective that woodland patch area, one of the most important variables in our study, is a variable that may mitigate negative effects of urbanization. To improve habitat suitability for woodpeckers, woodland patches should be large. This also suggests that large urban forests and parks may support more woodpeckers within towns which adds to the long debate on the functional role of size in urban parks [55], [56]. In the outskirts of cities there often occur large remnants of woodland patches which may be especially valuable woodpecker habitat requiring legal protection. Furthermore, woodlands should be multi-layered since this promotes species diversity in general [4], [56] and woodpecker species richness in particular. In cities this can be easily achieved by planting trees and shrubs in parks. Our results also indicate that increasing deciduous trees in forests may substantially improve their suitability for woodpeckers. The percentage of these trees should be higher in woodland patches in urbanized landscapes.
